# Triglyceride to high-density lipoprotein cholesterol ratio is associated with diabetes incidence in non-obese individuals with normoglycemia: a retrospective cohort study based on individuals from East Asia

**DOI:** 10.3389/fendo.2024.1442731

**Published:** 2024-11-11

**Authors:** Zhenhua Huang, Xigang Zhang, Dayong Sun, Ke Yu

**Affiliations:** ^1^ Department of Emergency Medicine, The First Affiliated Hospital of Shenzhen University, Shenzhen Second People’s Hospital, Shenzhen, China; ^2^ Department of Emergency Medicine, Pengpai Memorial Hospital, Shanwei, China; ^3^ Department of Gastroenterology, The First Affiliated Hospital of Shenzhen University, Shenzhen Second People’s Hospital, Shenzhen, China; ^4^ Department of Pulmonary and Critical Care Medicine, The First Affiliated Hospital of Shenzhen University, Shenzhen Second People’s Hospital, Shenzhen, China

**Keywords:** incident diabetes, TG/HDL-C ratio, non-obese, Chinese and Japanese, normoglycemia

## Abstract

**Background:**

Although several studies have explored the association between the triglyceride to high-density lipoprotein cholesterol ratio (TG/HDL-c) and diabetes risk, most of these studies are cross-sectional and typically involve small sample sizes, limiting the ability to draw causal inferences. Additionally, there is currently a few studies specifically focusing on non-obese individuals. Consequently, we conducted a retrospective cohort study to investigate the impact of TG/HDL-c on the risk of developing diabetes among non-obese, normoglycemic individuals across East Asian countries.

**Methods:**

This secondary retrospective cohort study recruited 85,029 non-obese individuals with normal glycemic levels from East Asian countries (China and Japan). We employed Cox proportional hazards regression models, incorporating cubic splines function for smooth curve fitting and using two-piecewise Cox regression for threshold effect analysis, to evaluate the nonlinear associations between baseline TG/HDL-c ratios and diabetes risk in non-obese individuals with normoglycemia. In addition, A range of subgroup and sensitivity analyses were performed to confirm the robustness of our results.

**Results:**

Among the individuals included, the average age was 42.14 ± 11.88 years, and 37,944 participants (44.62%) were male. After adjusting for covariates, the study revealed a significant correlation between the TG/HDL-c ratio and the risk of diabetes among non-obese individuals (HR=1.37, 95%CI: 1.22-1.54). Furthermore, a non-linear correlation was observed between the TG/HDL-c ratios and the incidence of non-obese diabetes, with an inflection point of 1.36. Under this threshold, the TG/HDL-c ratio notably boosts diabetes risk in non-obese populations, with an HR of 2.38 (95% CI: 1.57-3.59). Conversely, beyond the critical juncture, the upsurge in diabetes risk seems to level off, displaying no significant variation, with an HR of 1.18 (95% CI: 0.98-1.41).

**Conclusions:**

This study reveals a non-linear association between the TG/HDL-c ratios and the likelihood of diabetes in non-obese individuals from East Asia. Maintaining a ratio of TG/HDL-C below 1.36 significantly reduces diabetes risk. However, once the ratio of TG/HDL-C exceeds 1.36, reducing it does not substantially lower diabetes onset risk.

## Introduction

Diabetes mellitus (DM) is a chronic metabolic disorder and has emerged as a prevalent chronic condition globally. It continues to pose a significant health challenge on a global scale, impacting millions and leading to high rates of morbidity and mortality worldwide (1). The International Diabetes Federation (IDF) has documented that around 537 million adults received a diabetes diagnosis in 2021, a figure projected to increase to 643 million by 2030 and 783 million by 2045 (2). Diabetes is a heterogeneous disease closely associated with obesity. However, non-obese diabetes individuals are increasing, it has been observed that 10-20% of individuals with type diabetes mellitus (T2DM) are non-obese, particularly in Asian countries such as China, Singapore, and Japan, where the incidence of non-obese individuals account for as high as 60-80% of the total T2DM burden (3). This subgroup is often diagnosed later due to subtler symptoms compared to their obese counterparts and may experience complications earlier in the disease process, including cardiovascular diseases, neuropathy, and nephropathy. These unique epidemiological and clinical features highlight the need for tailored diagnostic and management strategies.

Dyslipidemia characterized by a disruption in blood lipid levels manifests as elevated triglycerides (TG) and low-density lipoprotein cholesterol (HDL-c), along with reduced high-density lipoprotein cholesterol (4). This imbalance is tightly linked to insulin resistance (IR), a crucial element in the progression from normoglycemia to diabetes. Research has shown that the triglycerides to high-density lipoprotein cholesterol(TG/HDL-c) ratio serves as a significant marker of insulin resistance (5, 6), where elevated ratios signal a heightened risk of diabetes and associated metabolic conditions. Therefore, it is essential to regularly monitor lipid levels to enable early detection and implement preventive measures in the care of individuals with diabetes.

It is well-established that obesity-related diabetes is primarily linked to IR and compromised insulin secretion (7, 8). However, understanding the pathophysiology of diabetes in non-obese individuals presents a challenge, as most existing models have focused on obese populations (3, 9, 10). The efficacy of using TG/HDL-c ratios as a predictor for diabetes in non-obese individuals remains undetermined, with prior research primarily consisting of cross-sectional or single-center studies. Consequently, we carried out a multi-center retrospective cohort study encompassing varying ethnicities (including Chinese and Japanese) to assess the impact of the TG/HDL-c ratio on diabetes progression in non-obese individuals. This novel approach not only fills gaps in our knowledge of diabetes pathophysiology in underrepresented populations but also seeks to increase the generalizability of findings across different demographic and ethnic groups. Ultimately, our goal is to develop targeted prevention strategies that are tailored to the specific needs of these diverse populations.

## Methods

### Study design

This study employed a retrospective cohort study design, with data sourced from the computer databases in China database established previously by Chinese researchers (Chen et al.) (11) and the NAGALA (NAfld in Gifu Area, Longitudinal Analysis) database from the Murakami Memorial Hospital in Japan (12). The TG/HDL-c ratio was an independent variable examined at baseline. The independent variable of interest was the incidence of DM among non-obese individuals with normoglycemia, categorized as a binary variable (0 = non-DM, 1 = DM).

### Data source

All data originated from the DATADRYAD database (www.datadryad.org). Chinese data were derived from a cohort study analyzing the correlation between body mass index, age, and diabetes onset among Chinese adults (Dryad dataset, accessible at https://doi.org/10.5061/dryad.ft8750v). Japanese data were drawn from a longitudinal study assessing ectopic fat obesity as a primary risk factor for T2DM among Japanese adults (Dryad dataset, accessible at https://doi.org/10.5061/dryad.8q0p192). Dryad’s usage policy permits the secondary analysis of these datasets without violating the original author’s rights.

### Study population

**Figure 1:**
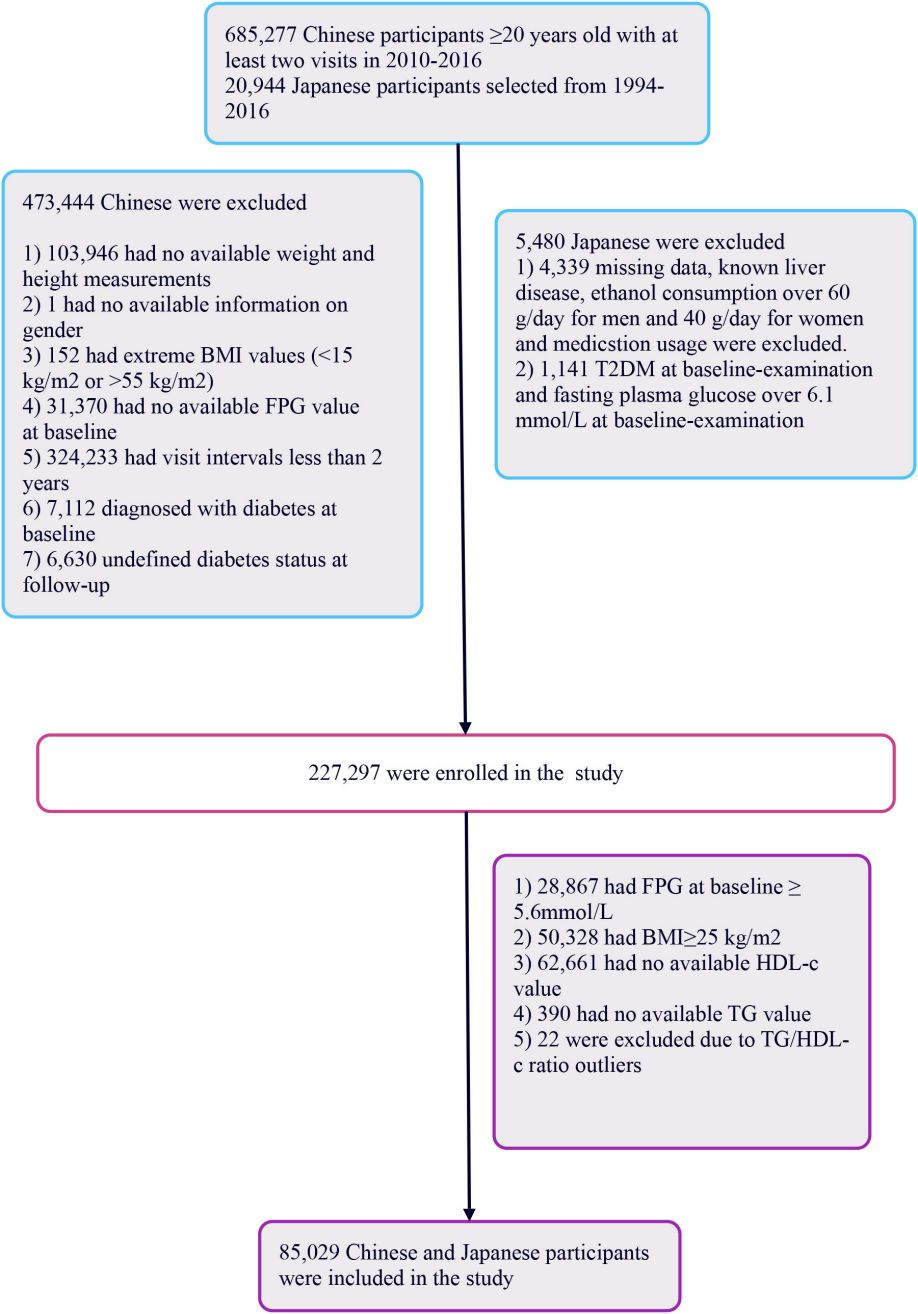
Flowchart of study participants.

The initial Chinese cohort comprised 685,277 participants, with 473,744 subsequently excluded, leaving 211,833 for analysis. The Japanese cohort began with 20,944 individuals, with 5,480 excluded, resulting in 15,464 participants analyzed. In total, 227,297 participants were included. Exclusion criteria for the study were: (i) participants with fasting blood glucose levels ≥5.6 mmol/L per 2021 American Diabetes Association standards were excluded (n=28,867), focusing on those with normal levels; (ii) individuals with a BMI ≥25 kg/m^2^ were also excluded (n=50,328), targeting non-obese individuals; (iii) participants lacking complete TG or HDL-c data, or those with abnormal or extreme ratios were excluded (n=63,073). This left 85,029 participants in the study (See **Figure 1**). This study followed the principles of the Declaration of Helsinki, with all protocols in accordance with applicable rules and regulations. As a secondary retrospective analysis, it did not require institutional ethical review or informed consent.

### Data collection

Data were gathered from cohorts from East Asian countries (China and Japan) (11, 12), documenting demographic characteristics (age, gender), fasting blood glucose (FBG), diastolic blood pressure (DBP), Total Cholesterol (TC), systolic blood pressure (SBP), HDL-c, TG, aspartate aminotransferase (AST), body mass index (BMI), and alanine aminotransferase (ALT). BMI calculations were based on height and weight, expressed as kg/m². Blood pressure was gauged using a standard mercury sphygmomanometer. Fasting venous blood samples were taken after a minimum fasting period of 10 hours. The selection of covariates was guided by clinical insights and literature, incorporating continuous variables like BMI, age, DBP, SBP, FBG, lipids (HDL-c, TC, TG), liver enzymes (ALT, AST), and a categorical variable for gender.

### Missing data processing

Missing data is inevitable in observational studies. In this research, missing data percentages were as follows: SBP and DBP both at 0.00% (8 cases each), TC at 0.00% (1 case), ALT at 0.33% (278 cases), and AST at 50.78% (43,179 cases). To address this issue, we utilized multiple imputation, incorporating linear regression across 10 iterations with variables like BMI, age, gender, DBP, SBP, AST, ALT, HDL-c, TG, and TC. This process assumed that the missing data were Missing at Random (MAR) (13).

### Statistical analysis

Participants were divided into four groups based on their TG/HDL-C ratios (14). For continuous variables, descriptive statistics were calculated using means and standard deviations for data that followed a normal distribution, or medians accompanied by interquartile ranges for data that were skewed. Categorical variables were described using frequencies and percentages. To evaluate the differences between TG/HDL-C ratio groups, several statistical methods were employed: χ2 tests were used for categorical variables, one-way ANOVA was applied for normally distributed data, and the Kruskal-Wallis H test was utilized for skewed data. Survival estimates and time-to-event data were calculated using the Kaplan-Meier method, and the differences in diabetes-free survival across the TG/HDL-C ratio groups were assessed with the log-rank test.

Utilizing both univariate and multivariate Cox proportional hazards models, we analyzed the correlation between the TG/HDL-C ratio and the risk of diabetes in non-obese participants. This analysis included a baseline unadjusted model, a minimally adjusted model (Model I: adjusted for gender and age), and a fully adjusted model (Model II: adjusted for gender, SBP, DBP, age, AST, BMI, ALT, TC, and baseline FPG). We computed hazard ratios (HR) and 95% confidence intervals (CI) to quantify effect sizes.

We employed Cox proportional hazards regression models, incorporating cubic splines function for smooth curve fitting to evaluate the nonlinear associations between baseline TG/HDL-c ratios and the risk of diabetes in non-obese individuals with normoglycemia (15). Then, we employed a two-piecewise Cox regression model for threshold effect analysis to further clarify this nonlinear association. By utilizing a log-likelihood ratio test, the most suitable model was determined for portraying the correlation between diabetes risk and the TG/HDL-c ratio. In addition, Various sensitivity and subgroup analyses were conducted to validate the strength of our findings. Initial analyses excluded participants over 60 years of age. Subsequent tests removed individuals with SBP ≥140 mmHg and explored the impact of excluding female participants from the model. Furthermore, we applied Generalized Additive Models (GAM) with continuous variables modeled as curves to enhance the robustness of our analytical approach.

We performed multiple subgroup analyses using a stratified Cox proportional hazards model, delineating strata by gender, age, BMI, SBP and DBP, and nationality. Initially, we transformed continuous variables such as age, BMI, SBP, and DBP into categories defined by clinical thresholds (Age categories: <30 years, 30-39 years, 40-49 years, 50-59 years, 60-69 years, 70-79 years, ≥80 years; BMI: <18.5 kg/m^2^, ≥18.5 kg/m^2^; SBP: <90 mmHg, ≥90 mmHg; SBP: <140 mmHg, ≥140 mmHg) (16). Subsequently, we adjusted for all remaining variables (age, gender, SBP, DBP, BMI, ALT, AST, TC, baseline FPG) within each stratified group. The presence of interaction terms was assessed using a likelihood ratio test to ensure comprehensive modeling.

The R software package (http://www.r-project.org, R Foundation) and Empower Stats (X&Y Solutions, Inc., Boston, MA, http://www.empowerstats.com) were utilized for performing statistical analyses. Statistical significance was determined by a P-value less than 0.05 (two-sided). The results adhere to STROBE guidelines (17).

## Results

### Characteristics of participants

**Figure 2:**
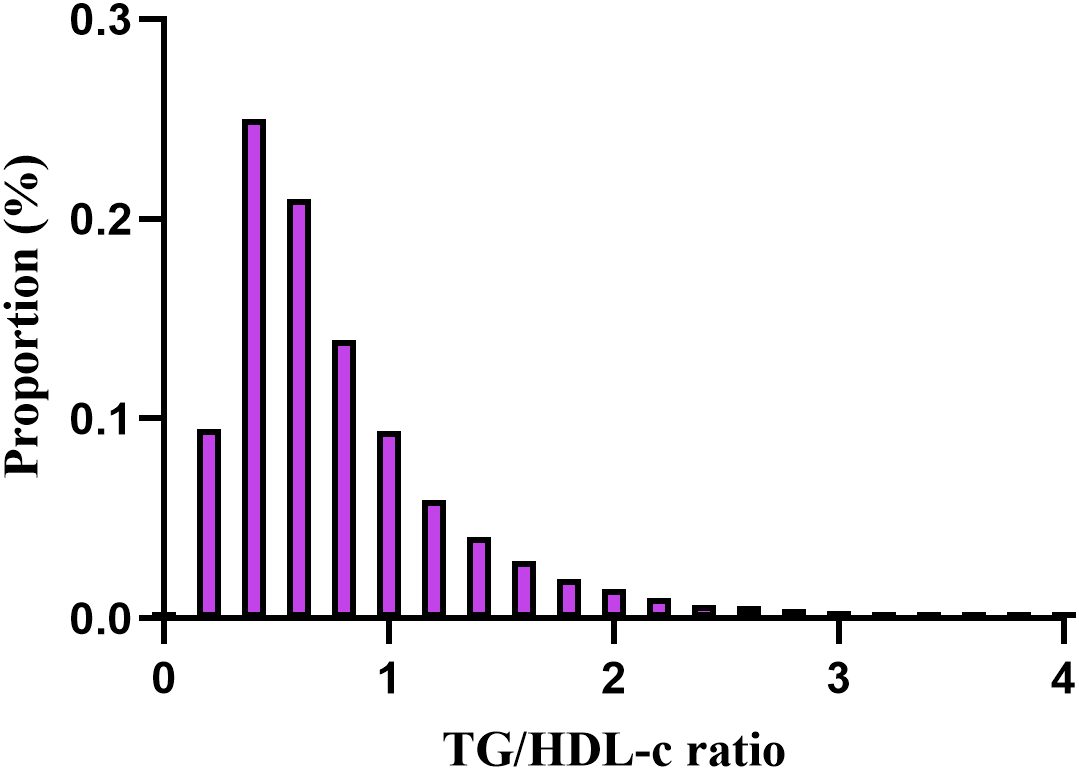
Distribution of TG/HDL-c ratio. It presented a skewed distribution, ranging from 0.025 to 9.829, with a mean of 0.834.

The demographic and clinical characteristics of the study participants are presented in **Table 1**, along with additional details provided in **Supplementary Tables 1**, **2**. The average age was 42.14 ± 11.88 years and 37,944 participants (44.62%) were male. During a median follow-up period of 3.02 years,393 individuals (0.46%) ultimately developed diabetes. The TG/HDL-c ratios varied between 0.025 and 9.829, with an average of 0.834 (see **Figure 2**). With the increasing TG/HDL-c quartiles, there was a significant upward trend in age, BMI, systolic and diastolic blood pressure (SBP and DBP), FBG, TC, TG, and liver enzymes (ALT and AST), accompanied by a decrease in HDL-c levels (all p-values < 0.001). The incidence of diabetes mellitus also increased significantly from Q1 to Q4 (0.24% to 0.91%, p < 0.001). A gender disparity was evident, with a higher proportion of males in the higher TG/HDL-c quartiles (**Table 1**).

**Table 1:** The baseline characteristics of participants.

TG/HDL-c ratio(quartile)	Q1 (≤0.42)	Q2 (0.43-0.64)	Q3 (0.65-1.00)	Q4 (>1.00)	P-value
participants	21,253	21,237	20,895	21,644	
Age (years)	39.38 ± 9.94	40.81 ± 11.46	42.78 ± 12.36	45.55 ± 12.64	<0.001
BMI (kg/m^2^)	20.54 ± 1.99	21.13 ± 2.02	21.76 ± 1.97	22.62 ± 1.68	<0.001
SBP (mmHg)	110.16 ± 13.47	113.29 ± 14.38	116.01 ± 15.09	119.83 ± 15.60	<0.001
DBP (mmHg)	68.52 ± 9.32	70.61 ± 9.60	72.26 ± 9.86	75.13 ± 10.21	<0.001
FBG (mg/dL)	4.76 ± 0.45	4.78 ± 0.46	4.81 ± 0.46	4.83 ± 0.48	<0.001
TC (mmol/L)	4.61 ± 0.81	4.61 ± 0.83	4.72 ± 0.87	4.97 ± 0.92	<0.001
TG (mmol/L)	0.52 ± 0.14	0.78 ± 0.15	1.08 ± 0.21	1.94 ± 0.92	<0.001
HDL-c (mmol/L)	1.69 ± 0.32	1.49 ± 0.26	1.37 ± 0.23	1.19 ± 0.23	<0.001
TG/HDL-c ratio	0.32 (0.26-0.38)	0.52 (0.47-0.58)	0.78 (0.71-0.88)	1.40 (1.16-1.87)	<0.001
ALT (U/L)	13.10 (10.70-17.30)	14.30 (11.00-19.50)	16.00 (12.10-22.60)	20.00 (15.00-28.90)	<0.001
AST (U/L)	19.21 ± 8.86	20.74 ± 11.75	21.86 ± 9.81	23.79 ± 16.50	<0.001
Sex					<0.001
Male	4,724 (22.23%)	7,802 (36.74%)	10,687 (51.15%)	14,731 (68.06%)	
Female	16,529 (77.77%)	13,435 (63.26%)	10,208 (48.85%)	6,913 (31.94%)	
Follow-up (year)	3.82 ± 2.37	3.44 ± 1.84	3.39 ± 1.71	3.40 ± 1.63	<0.001
Incident of DM	52 (0.24%)	63 (0.30%)	81 (0.39%)	197 (0.91%)	<0.001

Continuous variables were summarized as mean (SD) or medians (quartile interval); categorical variables were displayed as percentages (%). BMI, body mass index; SBP, systolic blood pressure; DBP, diastolic blood pressure; TC, total cholesterol; TG, triglyceride; HDL-c, high-density lipoprotein cholesterol; AST, aspartate aminotransferase; ALT, alanine aminotransferase; FBG, fasting plasma glucose; DM, diabetes mellitus.

Among Japanese participants, similar trends were observed, including notable increases in metabolic risk factors and DM incidence from Q1 to Q4. The follow-up duration was longer, suggesting a more extended period for potential DM development (**Additional File 1**: **Supplementary Table S1**). In addition, the trends among Chinese participants mirrored those of the overall population, showing significant escalations in metabolic risk factors and DM incidence rates across TG/HDL-c quartiles (**Additional File 1**: **Supplementary Table S2**).

### The association between TG/HDL-c ratio across quartiles and diabetes incidence

**Table 2** examined diabetes incidence across TG/HDL ratio quartiles, showing a trend of increasing incidence with higher quartiles (P < 0.001). Overall, the incidence was 0.46% (95% CI: 0.42-0.51), or 13.168 events/10,000 person-years. Incidence in Q1 was 0.24% (95% CI: 0.18-0.31), or 6.405 events/10,000 person-years, increasing in Q2 to 0.30% (95% CI: 0.22-0.37), or 8.624 events/10,000 person-years, in Q3 to 0.39% (95% CI: 0.30-0.47), or 11.435 events/10,000 person-years, and in Q4 to 0.91% (95% CI: 0.78-1.04), or 26.770 events/10,000 person-years.

**Table 2:** The Incidence rate of diabetes (Per 10,000 person-year).

(TG/HDL ratio quartiles)	Participants (n)	Diabetes events (n)	Incidence rate (95%CI) (%)	Per 10,000 person-year
Total	85,029	393	0.46 (0.42-0.51)	13.168
Q1	21,253	52	0.24 (0.18-0.31)	6.405
Q2	21,237	63	0.30(0.22-0.37)	8.624
Q3	20,895	81	0.39 (0.30-0.47)	11.435
Q4	21,644	197	0.91 (0.78-1.04)	26.770
P for trend				<0.001

**Figure 3:**
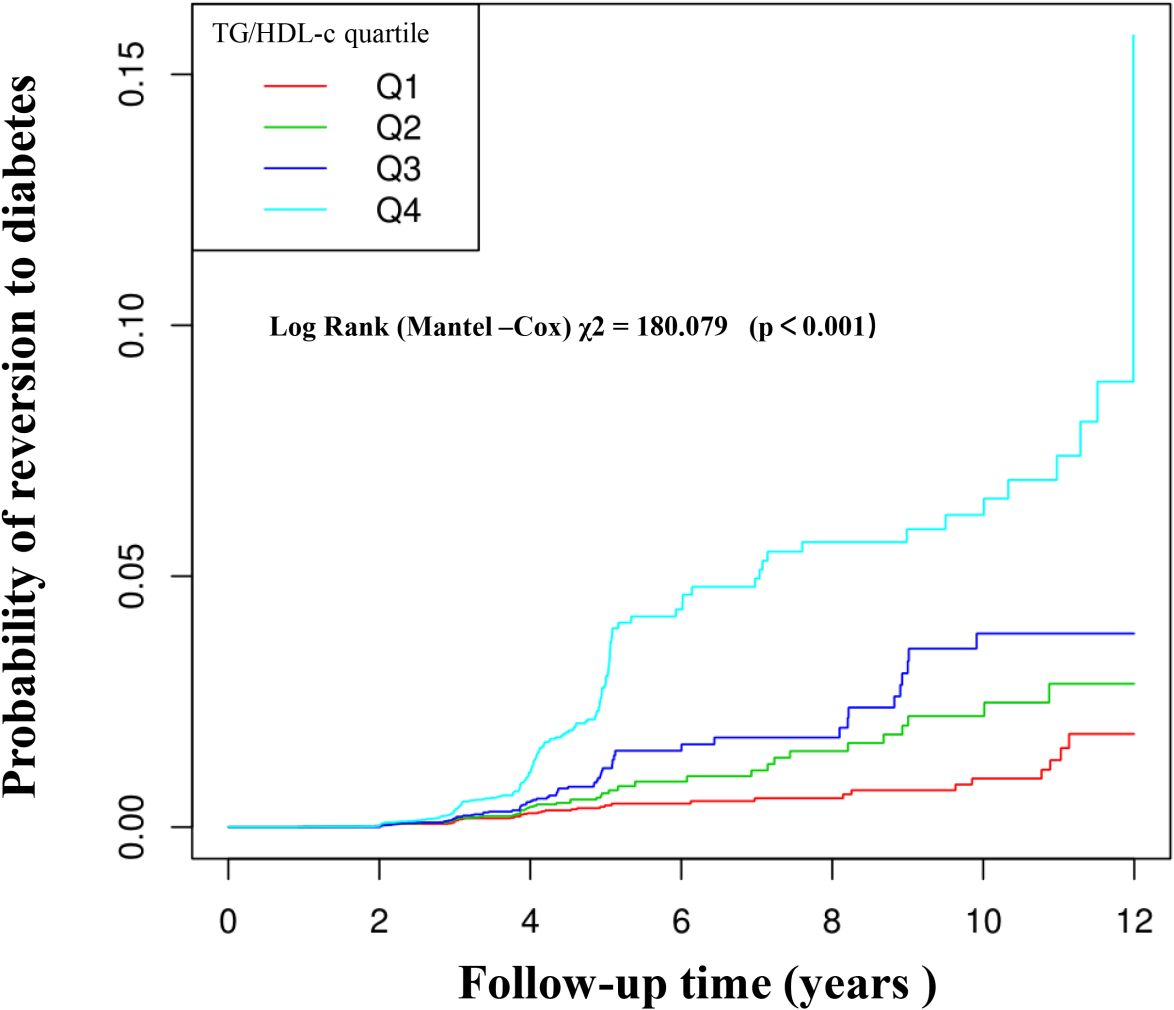
Kaplan–Meier curves for the probability of incident diabetes. The probability of incident diabetes increased progressively with a rising TG/HDL-c ratio, meaning that Patients with the highest TG/HDL-c ratio had a higher probability of reverting from normoglycemia to diabetes in non-obese individuals.

**Figure 3** illustrates Kaplan-Meier curves depicting the probability of t diabetes based on TG/HDL-c levels. The likelihood of incident diabetes significantly differed across TG/HDL-c groups (log-rank test, p<0.001). It steadily increased with higher TG/HDL-c ratios, indicating that individuals with the highest ratio had a greater probability of diabetes among non-obese individuals.

### Factors influencing the incident diabetes

The univariate analysis indicated positive associations between the diabetes risk and age, BMI, SBP, DBP, baseline FPG, ALT, AST, TG, TG/HDL-c ratio, and TC, all significant at P < 0.05. Conversely, a negative association was observed with HDL-c (all P < 0.05; detailed in **Table 3**).

**Table 3:** Univariate Cox proportional hazards regression was used to examine the factors influencing the incident diabetes in non-obese individuals.

Variable	Characteristics	HR (95% CI)	P-value
Age (years)	42.14 ± 11.88	1.09 (1.08, 1.09)	<0.0001
Sex			<0.0001
Male	37944 (44.62%)	1.0	
Female	47085 (55.38%)	0.65 (0.53, 0.80)	
BMI (kg/m2)	21.52 ± 2.07	1.31 (1.24, 1.38)	<0.0001
SBP (mmHg)	114.84 ± 15.09	1.04 (1.04, 1.05)	<0.0001
DBP (mmHg)	71.64 ± 10.05	1.05 (1.04, 1.06)	<0.0001
FPG (mmol/L)	86.79 ± 9.72	1.17 (1.16, 1.18)	<0.0001
TC (mmol/L)	4.73 ± 0.87	1.28 (1.15, 1.42)	<0.0001
TG (mmol/L)	1.09 ± 0.73	1.35 (1.31, 1.40)	<0.0001
HDL-c (mmol/L)	1.43 ± 0.32	0.45 (0.33, 0.62)	<0.0001
TG/HDL-c ratio	0.83 ± 0.69	1.60 (1.51, 1.70)	<0.0001
ALT (U/L)	15.80 (12.00-22.00)	1.00 (1.00, 1.00)	<0.0001
AST (U/L)	21.29 ± 12.05	1.01 (1.00, 1.01)	<0.0001

### The relationship between the TG/HDL-c ratio and the risk of diabetes

In analyzing the relationship between the TG/HDL-c ratio and the risk of diabetes, we employed multiple imputation to mitigate the impact of missing data on the results. We found that after imputation, the core results were consistent with those obtained from the real data (as shown in **Additional File 1**: **Supplementary Table S5**). Therefore, the results of this study are derived entirely from the analysis of the original data (real data). In this study, three models of Cox proportional hazards regression were used to assess the impact of the TG/HDL-c ratio on the transition from normoglycemia to diabetes. In the initial model without adjustments, a 1-unit elevation in the TG/HDL-c ratio resulted in a 60% increase in the chances of developing diabetes, with an HR of 1.60 (95% CI 1.51, 1.70, P<0.0001). The partially adjusted model, considering only age and gender, indicated that a 1-unit rise in the TG/HDL-c ratio was linked to a 47% higher probability of diabetes onset, with an HR of 1.47 (95% CI 1.37- 1.57, P<0.0001). The fully adjusted model demonstrated a 37% elevated risk of diabetes for each 1-unit increase in the TG/HDL-c ratio, yielding an HR of 1.37 (95% CI 1.22-1.54, P<0.0001). The consistent confidence intervals further support the strong relationship between the TG/HDL-c ratio and the development of diabetes, as illustrated in **Table 4**.

**Table 4:** Relationship between TG/HDL-c ratio and incident diabetes in non-obese individuals in different models.

Exposure	Crude model (HR,95%CI) P	Model I (HR,95%CI) P	Model II (HR,95%CI) P	Model III (HR,95%CI) P
TG/HDL-c ratio	1.60 (1.51, 1.70) <0.0001	1.47 (1.37, 1.57) <0.0001	1.37 (1.22, 1.54) <0.0001	1.33 (1.18, 1.50) <0.0001
(TG/HDL ratio quartiles)				
Q1	Ref	Ref	Ref	Ref
Q2	1.64 (1.14, 2.37) 0.0082	1.37 (0.94, 1.99) 0.0976	1.23 (0.76, 1.98) 0.3948	1.20 (0.74, 1.94) 0.4558
Q3	2.27 (1.60, 3.22) <0.0001	1.65 (1.15, 2.36) 0.0064	1.39 (0.87, 2.22) 0.1713	1.35 (0.84, 2.17) 0.2086
Q4	5.34 (3.92, 7.26) <0.0001	3.32 (2.40, 4.60) <0.0001	2.54 (1.63, 3.96) <0.0001	2.31 (1.47, 3.62) 0.0003
P for trend	<0.0001	<0.0001	<0.0001	<0.0001

Crude model: we did not adjust other covariates.

Model I: In the minimally adjusted model, we adjusted age and sex.

Model II: In the fully adjusted model, we adjusted age, sex, SBP, DBP, BMI, ALT, AST, TC, and FPG at baseline.

Model III: The generalized additive model (GAM), we adjusted age(smooth), sex, SBP (smooth), DBP (smooth), BMI (smooth), ALT (smooth), AST (smooth), TC (smooth), and FPG at baseline(smooth). HR, Hazard ratios; CI, confidence; Ref, reference.

Furthermore, we transformed the TG/HDL-c ratio into a categorical variable and reintroduced it into our model. The multivariate-adjusted model revealed that, compared to participants in the first quartile (Q1), those in the second to fourth quartiles (Q2-Q4) had HR of 1.23 (0.76-1.98), 1.39 (0.87-2.22), and 2.54 (1.63-3.96), respectively. This indicates a 23%, 39%, and 154% increased likelihood of progressing to diabetes for participants in Q2, Q3, and Q4, respectively (**Table 4**, Model II). When analyzing the data for Chinese and Japanese groups separately, the findings were consistent with those of the overall population (**Additional File 1**: **Supplementary Tables S3**, **S4**).

### Sensitivity analysis

We initiated our sensitivity analyses by employing the generalized additive model (GAM) in Model III, incorporating smoothing terms for additional variables. This model revealed a HR of 1.33 (95% CI: 1.18-1.50, P < 0.0001) (**Table 4**, Model III). After excluding participants above the age of 60 (8,399 individuals), adjustments were made for confounding variables, confirming a stable positive correlation between the TG/HDL-c ratio and diabetes progression, yielding a HR of 1.52 (95% CI: 1.43-1.63, P < 0.0001). Furthermore, eliminating participants with systolic blood pressure (SBP) of 140 mmHg or higher (4,869 individuals) and factoring in confounders revealed a consistent positive association, with an HR of 1.14 (95% CI: 1.31-1.52, P < 0.0001). An analysis that considered only male participants showed an HR of 1.38 (95% CI: 1.20-1.58, P < 0.0001). These extensive sensitivity analyses emphasize the reliability of our findings (**Table 5**).

**Table 5:** Relationship between TG/HDL-c ratio and incident diabetes in non-obese individuals in different sensitivity analyses.

Exposure	Model I (HR,95%CI) P	Model II(HR,95%CI) P	Model III (HR,95%CI) P
TG/HDL-c ratio	1.52 (1.43, 1.63) <0.0001	1.41 (1.31, 1.52) <0.0001	1.38 (1.20, 1.58) <0.0001
(TG/HDL-c ratio quartiles)			
Q1	Ref	Ref	Ref
Q2	1.27 (0.81, 1.99) 0.3054	1.05 (0.67, 1.66) 0.8247	1.11 (0.51, 2.44) 0.7945
Q3	1.90 (1.24, 2.89) 0.0029	1.33 (0.87, 2.04) 0.1921	1.34 (0.64, 2.80) 0.4403
Q4	4.34 (2.98, 6.34) <0.0001	2.54 (1.72, 3.75) <0.0001	2.46 (1.23, 4.91) 0.0105
P for trend	0.0002	0.0023	0.0005

The crude model I was a sensitivity analysis performed after excluding participants aged ≥60 years old (N= 8,399). we adjusted age, sex, SBP, DBP, BMI, ALT, AST, TC, and FPG at baseline.

Model II was a sensitivity analysis performed after excluding participants with SBP ≥140 mmHg (N= 4,869). we adjusted age, sex, SBP, DBP, BMI, ALT, AST, TC, and FPG at baseline.

Model III was a sensitivity analysis performed on participants without females (N= 47,085). we adjusted age, SBP, DBP, BMI, ALT, AST, TC, and FPG at baseline. HR, Hazard ratios; CI, confidence; Ref, reference.

### Employing cubic spline functions and the two-piecewise Cox proportional hazards regression model to address nonlinearity

**Figure 4:**
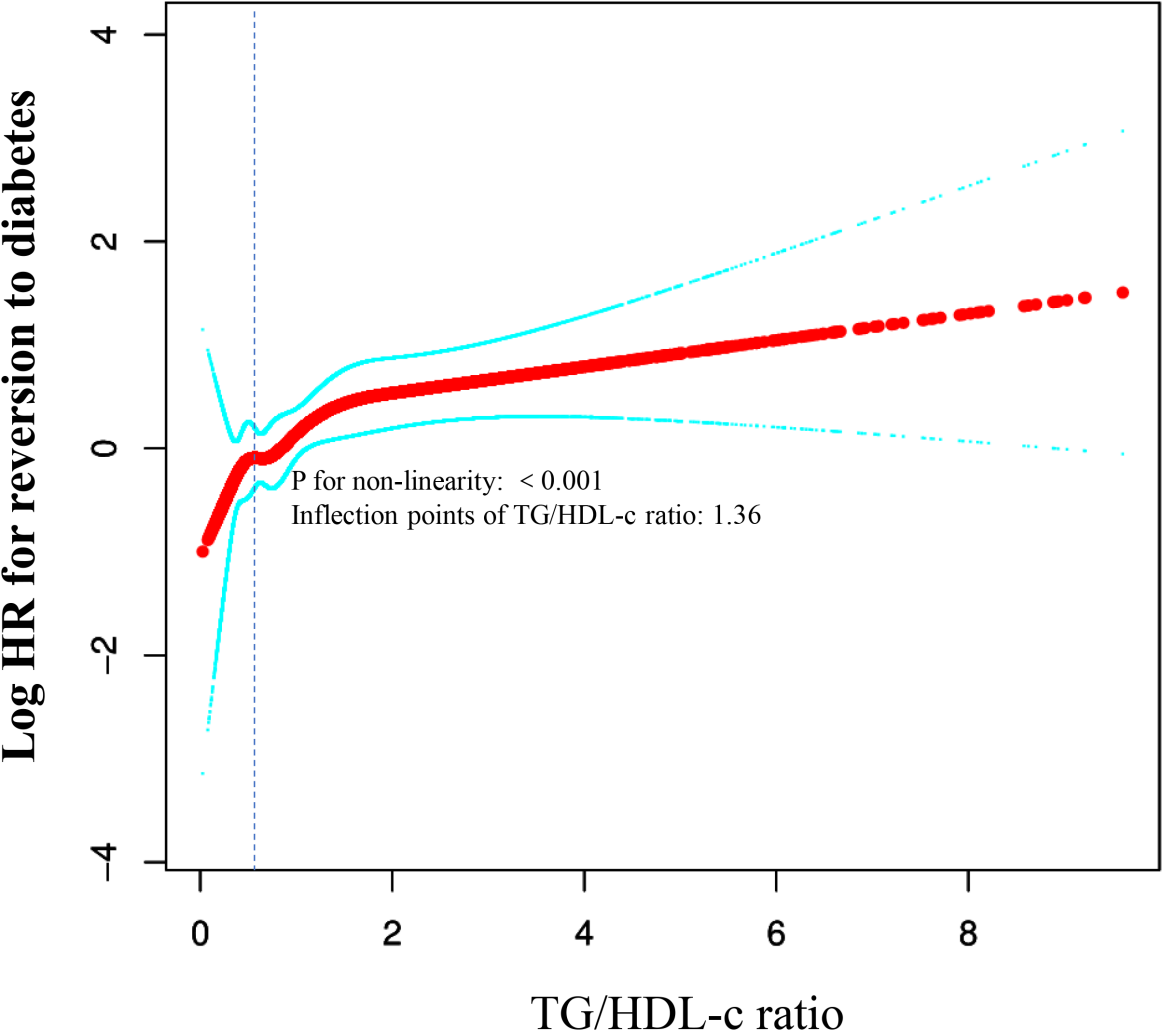
The non-linear relationship between TG/HDL-c and incident diabetes in non-obese individuals. The relationship between the TG/HDL-c ratio and the risk of diabetes was non-linear, with the inflection point of the TG/HDL-c ratio being 1.36. A non-linear relationship between them was detected after adjusting for age, sex, SBP, DBP, BMI, ALT, AST, TC, and FPG at baseline.

In the study, we discovered an inverted connection between TG/HDL-c ratios and the risk of diabetes in non-obese individuals across East Asian countries (China and Japan) (see **Figure 4**; **Table 6**). Initially, utilizing a Cox proportional hazards regression model with cubic spline functions, we examined this relationship and found it to be non-linear. Subsequently, we employed a two-segment Cox proportional hazards regression model to delve deeper into this association. The conventional Cox regression model unveiled a significant link between the TG/HDL-c ratio and the onset of diabetes, with an HR of 1.37 (95% CI: 1.22-1.54) and a P value of <0.0001. Furthermore, we pinpointed a critical point at 1.36 for the TG/HDL-c ratio. Below this threshold (<1.36), the HR sharply rose to 2.38 (95% CI: 1.57-3.59) with a P value of <0.0001. Conversely, for values equal to or exceeding the critical point (≥1.36), the HR stood at 1.18 (95% CI: 0.98-1.41) with a P value of 0.0742.

**Table 6:** The result of Two-piecewise Cox regression model.

Outcome: incident diabetes	HR, 95%CI	P -value
Standard Cox regression	1.37 (1.22, 1.54)	<0.0001
Two-piecewise Cox regression		
Inflection points of TG/HDL-c ratio	1.36	
<1.36	2.38 (1.57, 3.59)	<0.0001
≥1.36	1.18 (0.98, 1.41)	0.0742
P for log-likelihood ratio test	0.006	

We adjusted for age, sex, SBP, DBP, BMI, ALT, AST, TC, and FPG at baseline. HR, hazard ratios; CI, confidence.

### Subgroup analysis

The stratified analysis illustrates the results of a comprehensive subgroup analysis (**Table 7**). Age, BMI, gender, and systolic and diastolic blood pressures did not alter the association between the TG/HDL-c ratio and diabetes risk. Therefore, there were no significant interactions between these variables and the TG/HDL-c ratio (all interaction P values > 0.05). Additionally, we conducted subgroup analysis by nationality and found consistent results for both Chinese and Japanese populations.

**Table 7:** Stratified associations between TG/HDL-c ratio and diabetes risk by age, sex, BMI, SBP, DBP, and nation.

Variable	No of individuals	HR (95% CI)	P-value	P for interaction
**Age, yeas**				0.4203
<30	9,288	2.16 (0.68, 6.89)	0.1942	
30 to 40	33,733	1.26 (0.69, 2.31)	0.4466	
40 to 50	21,113	1.60 (1.33, 1.93)	<0.0001	
50 to 60	12,496	1.35 (1.14, 1.59)	0.0004	
60 to 70	6,092	1.19 (0.89, 1.60)	0.2442	
70 to 80	1,719	1.08 (0.56, 2.09)	0.8106	
≥80	588	0.56 (0.11, 2.91)	0.4907	
**BMI (kg/m ^2^)**				0.8309
<18.5	7,258	1.25 (0.35, 4.52)	0.7301	
≥18.5, <25	77,771	1.43 (1.28, 1.59)	<0.0001	
**Sex**				0.6473
Male	37,944	1.39 (1.22, 1.58)	<0.0001	
Female	47,085	1.31 (1.04, 1.65)	0.0217	
**SBP (mmHg)**				0.5863
<140	80,152	1.39 (1.22, 1.57)	<0.0001	
≥140	4,869	1.27 (0.95, 1.70)	0.1011	
**DBP (mmHg)**				0.9074
<90	81,124	1.38 (1.22, 1.56)	<0.0001	
≥90	3,897	1.35 (0.95, 1.91)	0.0897	
**Nation**				0.9959
Japanese	10,994	1.29 (1.02, 1.63)	0.0318	
Chinese	74,035	1.29 (1.13, 1.48)	0.0002	

The above model adjusted for age, sex, SBP, DBP, BMI, ALT, AST, TC, and FPG at baseline. In each case, the model is not adjusted for the stratification variable.

## Discussion

In this multi-center retrospective cohort study, we aimed to elucidate the association between the TG/HDL-c ratio and the risk of diabetes in non-obese individuals from East Asia. Our analysis shows that higher TG/HDL-c ratios significantly increase the risk of diabetes in non-obese Chinese and Japanese individuals with normoglycemia. Crucially, our results demonstrate a non-linear correlation, marked by a notable saturation effect in the independent relationship between the TG/HDL-c ratio and diabetes incidence in non-obese East Asian individuals. Specifically, while the hazard ratio increased significantly across the TG/HDL-c quartiles, peaking in the highest quartile with an adjusted HR of 2.54 (95% CI: 1.63-3.96), we identified an inflection point at a TG/HDL-c ratio of 1.36. Beyond this point, the increasing trend in diabetes risk appears to plateau, suggesting that interventions might be most critical and effective below this threshold. This saturation effect and the observed inflection point are pivotal for understanding the dynamic and complex nature of diabetes risk in non-obese individuals from East Asia, underscoring the need for targeted preventive strategies based on specific lipid profile patterns.

To the best of our knowledge, this is the inaugural study illustrating the influence of the TG/HDL-C ratio on the onset of T2DM across a broad spectrum of non-obese individuals from East Asia. A decade-long cohort study (18) in Japan revealed that for individuals with a BMI under 25 kg/m², the TG/HDL-C ratio predicted a higher risk for T2D (HR: 1.04 (95% CI 1.03–1.05)). For those with a BMI of 25 kg/m² or higher, the HR was 1.02 (95% CI 1.02–1.03), indicating an interaction between BMI and the TG/HDL-C ratio. This suggests different mechanisms of diabetes incidence in obese versus non-obese individuals. Thus, exploring the effect of the TG/HDL-C ratio on non-obese diabetes is warranted. In the current study, after accounting for confounders including age, sex, blood pressure, BMI, TC, FBG, ALT, and AST, we discovered that every single-unit rise in the TG/HDL-C ratio was associated with a 37% increase in the likelihood of incident diabetes, aligning with studies from South Korea and Singaporean Chinese populations (19–22). However, another study involving an Iranian population of 5064 subjects with a median follow-up period of 11.2 years yielded different results (23). Although this study indicated no direct association between the TG/HDL-C ratio and overall diabetes incidence, it highlighted a strong correlation with the progression in prediabetic individuals, suggesting a notable trend toward diabetes progression related to the TG/HDL-C ratio. It is inferred that potential factors contributing to these discrepancies include a smaller study cohort (only 360 individuals progressed to diabetes) and possibly slower rates of progression from normoglycemia to diabetes, underscoring pronounced ethnic variations in the development of diabetes. Moreover, one reason to consider is that the study focused exclusively on non-obese individuals. Research indicates that genetic factors, which vary significantly across regions, are the strongest indicators of T2DM in non-obese populations. China and Japan, both predominantly East Asian populations, share similar genetic structures (3). Our study conducted subgroup analyses comparing Chinese and Japanese cohorts, yielding consistent conclusions further validating the influence of genetics on diabetes. This underscores the importance of considering ethnic and regional variations when studying the pathogenesis and progression of diabetes in non-obese populations. Most importantly, our study primarily demonstrates a non-linear association between the TG/HDL-C ratio and diabetes risk. Notably, maintaining a ratio of TG/HDL-C below 1.36 significantly reduces diabetes risk. However, once the ratio of TG/HDL-C exceeds 1.36, reducing it does not substantially lower diabetes onset risk, highlighting the need to manage other risk factors Such as weight management, dietary adjustments, quitting alcohol and smoking, as well as prevention and treatment of cardiovascular diseases. Furthermore, our findings suggest that this non-linear relationship, while potentially unpredictable, may more accurately reflect the true dynamics between these variables. It is noteworthy that we also found a saturation effect in the relationship between the TG/HDL-C ratio and diabetes risk. Specifically, maintaining a TG/HDL-C ratio below 1.36 significantly reduces the risk of diabetes. However, once the TG/HDL-C ratio exceeds 1.36, lowering this ratio does not significantly decrease the risk of diabetes onset, highlighting the necessity of managing other risk factors, such as weight management, dietary adjustments, cessation of alcohol and smoking, and the prevention and treatment of cardiovascular diseases. This conclusion is consistent with the findings of Sun Y et al. (24), who focused on a prediabetic Chinese population and identified a non-linear relationship and saturation effect between the TG/HDL-C ratio and diabetes risk. However, their inflection point was 1.415, which is higher than our established point, suggesting that the TG/HDL-C ratio may have a greater impact on patients with prediabetes. Nevertheless, our study contrasts with some other research findings. For example, Wang H et al. (14) analyzed the relationship between the TG/HDL-C ratio and type 2 diabetes risk in a cohort of 15,443 Japanese individuals, revealing a non-linear relationship with a threshold effect: when the TG/HDL-C ratio exceeds 0.35, the risk of diabetes increases by 20%. Our findings are consistent with those of Sun Y et al., while they differ from the conclusions of Wang H et al. Nonetheless, our sample size is several times larger than that of Wang H et al.’s study, leading us to believe that the conclusions drawn from our study may be more accurate.

Diabetes is marked by increased hepatic glucose output, insulin resistance, and insufficient insulin secretion (25). Research has extensively documented insulin resistance compounded by defective insulin secretion in obese individuals with T2DM, yet the pathophysiology in non-obese individuals is not as well understood due to the focus predominantly on obese populations. However, emerging studies suggest that non-obese individuals may display metabolic obesity characteristics, pointing to similar underlying pathophysiological mechanisms. The TG/HDL-c ratio is positively correlated with insulin resistance, which is known to predict an approximately 80% increased risk of diabetes in non-obese individuals (26). HDL-c is believed to mitigate insulin resistance by counteracting the effects of LDL-C, which reduces the expression of cyclin B1 in pancreatic β-cells and thereby exacerbates insulin resistance (27). Additionally, HDL may regulate glucose homeostasis through mechanisms such as enhancing insulin secretion, facilitating direct glucose uptake by muscles, and boosting insulin sensitivity (28, 29). Conversely, hypertriglyceridemia raises free fatty acid levels, which promote insulin resistance. Accumulation of these acids in pancreatic islets can lead to β-cell dysfunction and apoptosis (30, 31). Therefore, the TG/HDL-c ratio is a more predictive marker for the development of diabetes than either HDL-C or TG levels alone.

Our study had some strengths: The inclusion of large, long-term cohort studies from East Asian countries (specifically China and Japan) ensures a significant sample size and prolonged observation period, which enhances the reliability, depth, and overall comprehensiveness of our findings (2). To the best of our knowledge, this is the initial study to explore the influence of the TG/HDL-C ratio on T2DM progression among diverse non-obese East Asian individuals (3). This investigation revealed a nonlinear association between the TG/HDL-c ratio and diabetes vulnerability, highlighting a critical inflection point. Exploring this link further can refine our approach to early intervention in dyslipidemia-related diabetes risks (4). To tackle missing data, we utilized multiple imputations, a method designed to maximize statistical power and reduce potential biases associated with incomplete covariate information (5). A range of sensitivity analyses were performed to confirm the robustness of our findings. Initially, we reanalyzed the relationship between the TG/HDL-c ratio and diabetes risk by separately excluding individuals with SBP ≥140 mmHg, those over 60 years of age, and female participants. Subsequently, we employed a generalized additive model (GAM) with continuous variables modeled as curves to further explore the association (6). We conducted detailed subgroup analyses, adjusting for age, blood pressure, and gender, which consistently showed a positive association between the TG/HDL-c ratio and diabetes risk susceptibility across various subgroups, reinforcing the reliability of our findings.

Despite the strengths mentioned above, the study also presents certain limitations: First, as all participants were of East Asian descent, encompassing Chinese and Japanese individuals, additional research is essential to explore the impact of the TG/HDL-c ratio on diabetes risk across varied genetic profiles. Secondly, this analysis is based on secondary retrospective data, and due to the limitations of the original dataset, adjustments for variables such as insulin concentration were not possible. Additionally, some covariates, including dietary habits and exercise levels, were unavailable for analysis. Moreover, a considerable number of participants lost to follow-up rate was observed in this study. Future research should consider conducting another prospective cohort study or randomized controlled trial to mitigate potential biases. Thirdly, some data were missing, which affected the integrity of the dataset and may have introduced bias. However, we employed methods such as multiple imputation to further verify the robustness of the results. Fourthly, this study defined diabetes based on fasting plasma glucose levels equal to or greater than 7.00 mmol/l, or self-reported diabetes status. However, it did not employ a 2-hour oral glucose tolerance test or measure glycated hemoglobin levels, potentially underestimating diabetes incidence. Fourth, TG and HDL-cholesterol levels were measured only at baseline in the original study. Future studies should monitor the temporal variations of these lipid profiles to capture dynamic changes.

## Conclusions

Our study confirms a curvilinear association between the TG/HDL-c ratio and diabetes risk in non-obese individuals from East Asia (specifically China and Japan), with a significant inflection point at 1.36. Maintaining a ratio of TG/HDL-C below 1.36 significantly reduces diabetes risk. However, once the ratio of TG/HDL-C exceeds 1.36, reducing it does not substantially lower diabetes onset risk. These findings highlight the necessity for tailored interventions in non-obese individuals to preemptively reduce diabetes risk.

## Data Availability

The datasets presented in this study can be found in online repositories. The names of the repository/repositories and accession number(s) can be found in the article/**Supplementary Material**.
