# Supplementary material for: Triglyceride to high-density lipoprotein cholesterol ratio is associated with diabetes incidence in non-obese individuals with normoglycemia: a retrospective cohort study based on individuals from East Asia
Source: Front Endocrinol (Lausanne). 2024 Nov 11;15:1442731. doi: 10.3389/fendo.2024.1442731 (PMC11586199; doi:10.3389/fendo.2024.1442731)
Supplement: Supplementary file 1 [file DataSheet1.docx]

[Additional file 1](https://static-content.springer.com/esm/art%3A10.1186%2Fs12933-023-01762-2/MediaObjects/12933_2023_1762_MOESM1_ESM.docx)

**Table S1.**The baseline characteristics of Japanese participants.

| TG/HDL-c ratio (quartile) | Q1 (≤0.42) | Q2 (0.43-0.64) | Q3 (0.65-1.00) | Q4 (＞1.00) | P-value |
| --- | --- | --- | --- | --- | --- |
| participants | 5,477 | 2,266 | 1,760 | 1,491 |  |
| Age (years) | 41.34 ± 8.33 | 44.00 ± 8.97 | 44.80 ± 9.08 | 45.78 ± 8.79 | <0.001 |
| BMI (kg/m2) | 20.23 ± 2.04 | 21.14 ± 2.00 | 21.70 ± 1.93 | 22.39 ± 1.72 | <0.001 |
| SBP (mmHg) | 107.70 ± 12.24 | 112.11 ± 12.91 | 113.71 ± 12.95 | 116.92 ± 13.62 | <0.001 |
| DBP (mmHg) | 66.72 ± 8.76 | 69.91 ± 9.12 | 71.38 ± 9.17 | 73.89 ± 9.63 | <0.001 |
| FBG (mg/dL) | 4.93 ± 0.33 | 5.04 ± 0.32 | 5.10 ± 0.29 | 5.13 ± 0.28 | <0.001 |
| TC (mmol/L) | 4.88 ± 0.80 | 5.04 ± 0.85 | 5.17 ± 0.82 | 5.38 ± 0.86 | <0.001 |
| TG (mmol/L) | 0.46 ± 0.15 | 0.75 ± 0.16 | 1.01 ± 0.21 | 1.69 ± 0.70 | <0.001 |
| HDL-c (mmol/L) | 1.76 ± 0.37 | 1.46 ± 0.27 | 1.29 ± 0.24 | 1.09 ± 0.24 | <0.001 |
| TG/HDL-c ratio | 0.27 (0.20-0.34) | 0.51 (0.47-0.57) | 0.78 (0.70-0.87) | 1.38 (1.16-1.84) | <0.001 |
| ALT (U/L) | 14.00 (11.00-18.00) | 15.00 (12.00-20.00) | 17.00 (13.00-23.00) | 20.00 (15.00-26.00) | <0.001 |
| AST (U/L) | 17.00 ± 6.28 | 17.18 ± 5.36 | 18.00 ± 5.85 | 19.51 ± 16.50 | <0.001 |
| Sex |  |  |  |  | <0.001 |
| Male | 1439 (26.27%) | 1180 (52.07%) | 1193 (67.78%) | 1220 (81.82%) |  |
| Female | 4038 (73.73%) | 1086 (47.93%) | 567 (32.22%) | 271 (18.18%) |  |
| Follow-up (year) | 5.81 ± 3.69 | 6.36 ± 3.82 | 6.53 ± 3.82 | 6.76 ± 3.82 | <0.001 |
| Incident of MD | 20 (0.37%) | 18 (0.79%) | 14 (0.80%) | 26 (1.74%) | <0.001 |

**Table S2.** The baseline characteristics of Chinese participants.

| TG/HDL-c ratio (quartile) | Q1 (≤0.42) | Q2 (0.43-0.64) | Q3 (0.65-0.99) | Q4 (≥1.00) | P-value |
| --- | --- | --- | --- | --- | --- |
| participants | 15776 | 18971 | 19135 | 20153 |  |
| Age (years) | 38.70 ± 10.36 | 40.43 ± 11.67 | 42.59 ± 12.60 | 45.54 ± 12.88 | <0.001 |
| BMI (kg/m2) | 20.64 ± 1.97 | 21.13 ± 2.02 | 21.76 ± 1.97 | 22.64 ± 1.68 | <0.001 |
| SBP (mmHg) | 111.01 ± 13.77 | 113.43 ± 14.54 | 116.22 ± 15.26 | 120.05 ± 15.71 | <0.001 |
| DBP (mmHg) | 69.15 ± 9.42 | 70.69 ± 9.65 | 72.34 ± 9.92 | 75.22 ± 10.24 | <0.001 |
| FBG (mg/dL) | 4.70 ± 0.46 | 4.74 ± 0.47 | 4.78 ± 0.46 | 4.81 ± 0.49 | <0.001 |
| TC (mmol/L) | 4.51 ± 0.79 | 4.56 ± 0.81 | 4.68 ± 0.86 | 4.94 ± 0.92 | <0.001 |
| TG (mmol/L) | 0.54 ± 0.13 | 0.79 ± 0.15 | 1.09 ± 0.21 | 1.96 ± 0.93 | <0.001 |
| HDL-c (mmol/L) | 1.66 ± 0.30 | 1.50 ± 0.26 | 1.38 ± 0.23 | 1.20 ± 0.23 | <0.001 |
| TG/HDL-c ratio | 0.34 (0.29-0.38) | 0.53 (0.47-0.58) | 0.78 (0.71-0.88) | 1.40 (1.16-1.88) | <0.001 |
| ALT (U/L) | 13.00 (10.50-17.20) | 14.10 (11.00-19.40) | 16.00 (12.00-22.50) | 20.10 (14.90-29.00) | <0.001 |
| AST (U/L) | 21.09 ± 10.21 | 21.75 ± 12.82 | 22.70 ± 10.28 | 24.57 ± 16.38 | <0.001 |
| Sex |  |  |  |  | <0.001 |
| Male | 3285 (20.82%) | 6622 (34.91%) | 9494 (49.62%) | 13511 (67.04%) |  |
| Female | 12491 (79.18%) | 12349 (65.09%) | 9641 (50.38%) | 6642 (32.96%) |  |
| Follow-up (year) | 3.14 ± 0.98 | 3.09 ± 0.95 | 3.11 ± 0.93 | 3.15 ± 0.94 | <0.001 |
| Incident of MD | 32 (0.20%) | 45 (0.24%) | 67 (0.35%) | 171 (0.85%) | <0.001 |

**Table S3.** Relationship between TG/HDL-c ratio and transition to diabetes from normoglycemia in non-obese Japanese individuals in different models

| Exposure | Crude model (HR,95%CI) P | Model I(HR,95%CI) P | Model II(HR,95%CI) P |
| --- | --- | --- | --- |
| TG/HDL-c ratio | 1.66 (1.37, 2.03) <0.0001 | 1.48 (1.19, 1.84) 0.0004 | 1.30 (1.01, 1.67) 0.0414 |
| (TG/HDL ratio quartiles) |  |  |  |
| Q1 | Ref | Ref | Ref |
| Q2 | 1.88 (0.99, 3.55) 0.0528 | 1.51 (0.78, 2.89) 0.2185 | 1.25 (0.64, 2.42) 0.5116 |
| Q3 | 1.80 (0.91, 3.58) 0.0904 | 1.38 (0.68, 2.82) 0.3723 | 1.04 (0.50, 2.16) 0.9082 |
| Q4 | 3.76 (2.10, 6.75) <0.0001 | 2.77 (1.46, 5.28) 0.0019 | 1.81 (0.90, 3.62) 0.0948 |
| P for trend | <0.0001 | 0.0036 | 0.1401 |

Crude model: we did not adjust other covariates.

Model I: we adjusted age and sex.

Model II: we adjusted age, sex, SBP, DBP, BMI, ALT, AST, TC, and FPG at baseline.

**Table S4.** Relationship between TG/HDL-c ratio and transition to diabetes from normoglycemia in non-obese Chinese individuals in different models

| Exposure | Crude model (HR,95%CI) P | Model I(HR,95%CI) P | Model II(HR,95%CI) P |
| --- | --- | --- | --- |
| TG/HDL-c ratio | 1.52 (1.43, 1.63) <0.0001 | 1.41 (1.31, 1.52) <0.0001 | 1.27 (1.10, 1.45) 0.0009 |
| (TG/HDL ratio quartiles) |  |  |  |
| Q1 | Ref | Ref | Ref |
| Q2 | 1.27 (0.81, 1.99) 0.3054 | 1.05 (0.67, 1.66) 0.8247 | 1.04 (0.51, 2.10) 0.9220 |
| Q3 | 1.90 (1.24, 2.89) 0.0029 | 1.33 (0.87, 2.04) 0.1921 | 1.25 (0.65, 2.42) 0.5026 |
| Q4 | 4.34 (2.98, 6.34) <0.0001 | 2.54 (1.72, 3.75) <0.0001 | 2.06 (1.10, 3.84) 0.0233 |
| P for trend | <0.0001 | 0.0036 | 0.0023 |

Crude model: we did not adjust other covariates.

Model I: we adjusted age and sex.

Model II: we adjusted age, sex, SBP, DBP, BMI, ALT, AST, TC, and FPG at baseline.

**Table S5.** Relationship between TG/HDL-c ratio and incident diabetes in non-obese individuals in different models by Multiple Imputation data.

| Exposure | Crude model (HR,95%CI) P | Model I  (HR,95%CI) P | Model II  (HR,95%CI) P |
| --- | --- | --- | --- |
| TG/HDL-c ratio | 1.60 (1.51, 1.70) <0.0001 | 1.47 (1.37, 1.57) <0.0001 | 1.40 (1.30, 1.51) <0.0001 |
